# Deficit irrigation combined with straw mulching maintains maize yield and improves soil quality in semi-arid environments

**DOI:** 10.3389/fpls.2026.1823857

**Published:** 2026-06-15

**Authors:** Shumiao Lu, Bing Xu, Delong Tian, Guoshuai Wang, Yanwei Liu

**Affiliations:** 1 Institute of Pastoral Hydraulic Research, Hohhot, China; 2 Yinshanbeilu Grassland Eco-Hydrology National Observation and Research Station, China Institute of Water Resources and Hydropower Research, Beijing, China; 3 Faculty of Modern Agricultural Engineering, Kunming University of Science and Technology, Kunming, China

**Keywords:** deficit irrigation, maize yield, soil enzyme activity, soil nutrients, soil organic carbon, soil quality, straw mulching

## Abstract

**Introduction:**

Water scarcity severely limits maize production in semi-arid environments, making it essential to develop water saving management strategies that maintain soil quality and crop productivity.

**Methods:**

A field experiment was conducted from May to October 2024 in West Ordos, Inner Mongolia, China, using three deficit irrigation levels (W1: 2625, W2: 2400, and W3: 2175 m³ ha⁻¹) and two straw mulching treatments (F1: 9000 kg ha⁻¹ and F2: no mulching) arranged in a randomized block design. Soil nutrients, enzyme activities, soil organic carbon fractions, soil quality index (SQI), maize yield, water use efficiency (WUE), and irrigation water use efficiency (IWUE) were evaluated.

**Results:**

Increasing deficit irrigation intensity reduced soil nutrients, several enzyme activities, labile organic carbon fractions, and maize yield, whereas straw mulching partly alleviated these negative effects and improved integrated soil quality. Straw mulching improved SQI across deficit irrigation levels, and W2F1 maintained a grain yield comparable to W1F1 while achieving the highest WUE and IWUE. Correlation analysis showed that maize yield was positively correlated with key soil quality indicators, including phosphatase, coarse particulate organic carbon, sucrase, and SQI. Structural equation modeling showed good model fit and explained 72.6% of the variation in maize yield, suggesting that yield responses were associated with both the direct effects of deficit irrigation and mulching management and changes in enzyme activity and organic carbon fractions.

**Discussion:**

W1F1 showed the highest integrated soil quality, whereas W2F1 provided the best balance between yield maintenance, water saving, and soil quality preservation, indicating that moderate deficit irrigation combined with straw mulching is a feasible strategy for sustainable maize production in semi-arid agroecosystems.

## 1 Introduction

Maize (Zea mays L.) is one of the most widely cultivated cereal crops worldwide (Revilla et al., 2022) and contributes substantially to global food and feed security (Kaul et al., 2019). However, in semi-arid environments, maize production is severely constrained by limited water availability and increasing soil degradation (Wang et al., 2021). In areas such as West Ordos, Inner Mongolia, precipitation is low and unevenly distributed, making crop growth highly dependent on irrigation (Ye et al., 2023). Excessive irrigation, although effective in maintaining yield in the short term, intensifies pressure on groundwater resources and threatens the sustainability of agricultural systems (Fereres and Soriano, 2007). Therefore, developing water saving management strategies that can maintain crop productivity while improving soil quality has become a major challenge for sustainable maize production in semi-arid agroecosystems (Melkie et al., 2024; Zhang et al., 2024).

Deficit irrigation and straw mulching have been widely adopted as water saving practices in semi-arid agricultural systems (Farré and Faci, 2009; Zhang et al., 2015). Moderate deficit irrigation can reduce irrigation input while maintaining relatively stable yields and improving water use efficiency. For instance, Melkie et al. (2024) reported that maize yield decreased by 8-13.5% under deficit irrigation accompanied by an improvement in water use efficiency. However, the yield response to deficit irrigation varies depending on irrigation intensity, growth stage, and environmental conditions (El-Sanatawy et al., 2021; Bian et al., 2024). Under additional stress such as salinity, yield reductions may be further aggravated (Kaul et al., 2019; Gao et al., 2025). In addition to yield responses, irrigation regimes can alter soil nutrient availability and enzyme activity, thereby influencing soil biological processes (Muhammad et al., 2022).

Straw mulching is recognized as an effective management strategy to conserve soil moisture, reduce evaporation, regulate soil temperature, and increase organic matter inputs (Lal, 2015; Qin et al., 2015). A five-year field experiment conducted by Wang et al. (2018) on the Loess Plateau under dryland conditions demonstrated that straw mulching increased maize grain yield by 5-33% and aboveground biomass by 8-15% compared with no mulching treatment, while significantly enhancing soil organic carbon fractions. Therefore, integrating deficit irrigation with straw mulching may provide a promising approach to balancing water conservation and soil quality improvement in semi-arid environments.

Soil quality is a critical determinant for sustaining crop growth and regulating nutrient cycling. It is commonly evaluated using indicators, including soil organic carbon fractions, soil enzyme activities, and nutrient status, which are commonly incorporated into soil quality assessment systems (Li et al., 2021). In semi-arid environments, the combined effects of straw mulching and deficit irrigation can influence soil carbon transformation processes, enzymatic reactions, and nutrient availability, ultimately impacting crop yield formation (Yang et al., 2023; Cheng et al., 2025). Although previous studies have confirmed the individual benefits of deficit irrigation and straw mulching (Tao et al., 2015; Yu et al., 2018; Cheng et al., 2021; Zou et al., 2021), limited research has quantified their combined effects on soil quality and maize yield under semi-arid conditions. In particular, most existing studies focus on the isolated responses of crop yield or soil physicochemical properties, whereas the interactions among soil organic carbon fractions, soil enzyme activities, and soil nutrients remain insufficiently understood.

In summary, this study was conducted in a maize field in West Ordos, Inner Mongolia, to evaluate the interactive effects of different deficit irrigation levels and straw mulching on soil organic carbon fractions, enzyme activities, nutrient status, and maize yield. By integrating correlation analysis, principal component analysis, and structural equation modeling, this study aims to clarify the relationships among soil quality indicators and their association with yield formation, thereby providing a scientific basis for sustainable water saving maize production in semi-arid agroecosystems.

## 2 Materials and methods

### 2.1 Experimental site

**Figure 1:**
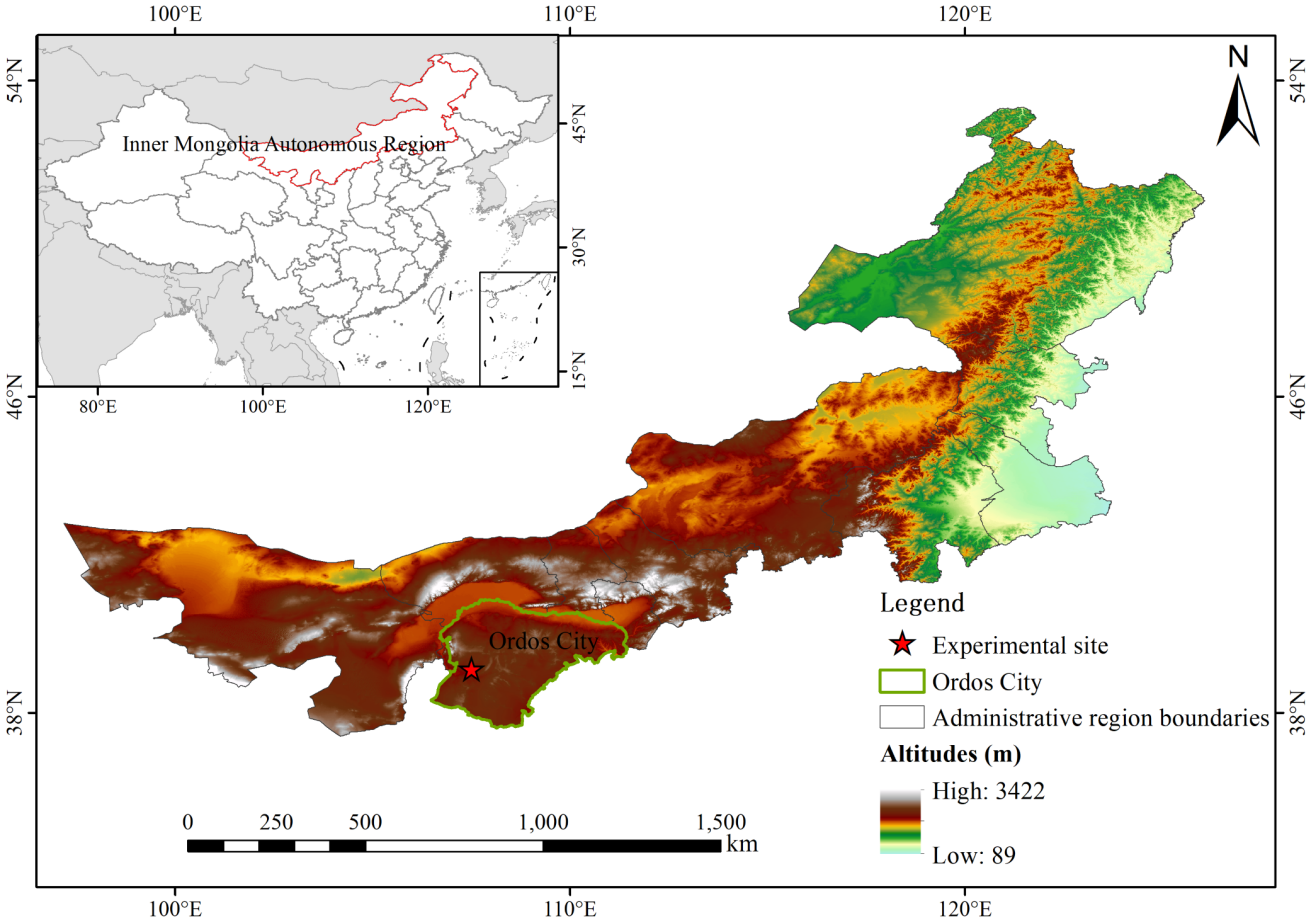
Location of the study area.

**Figure 2:**
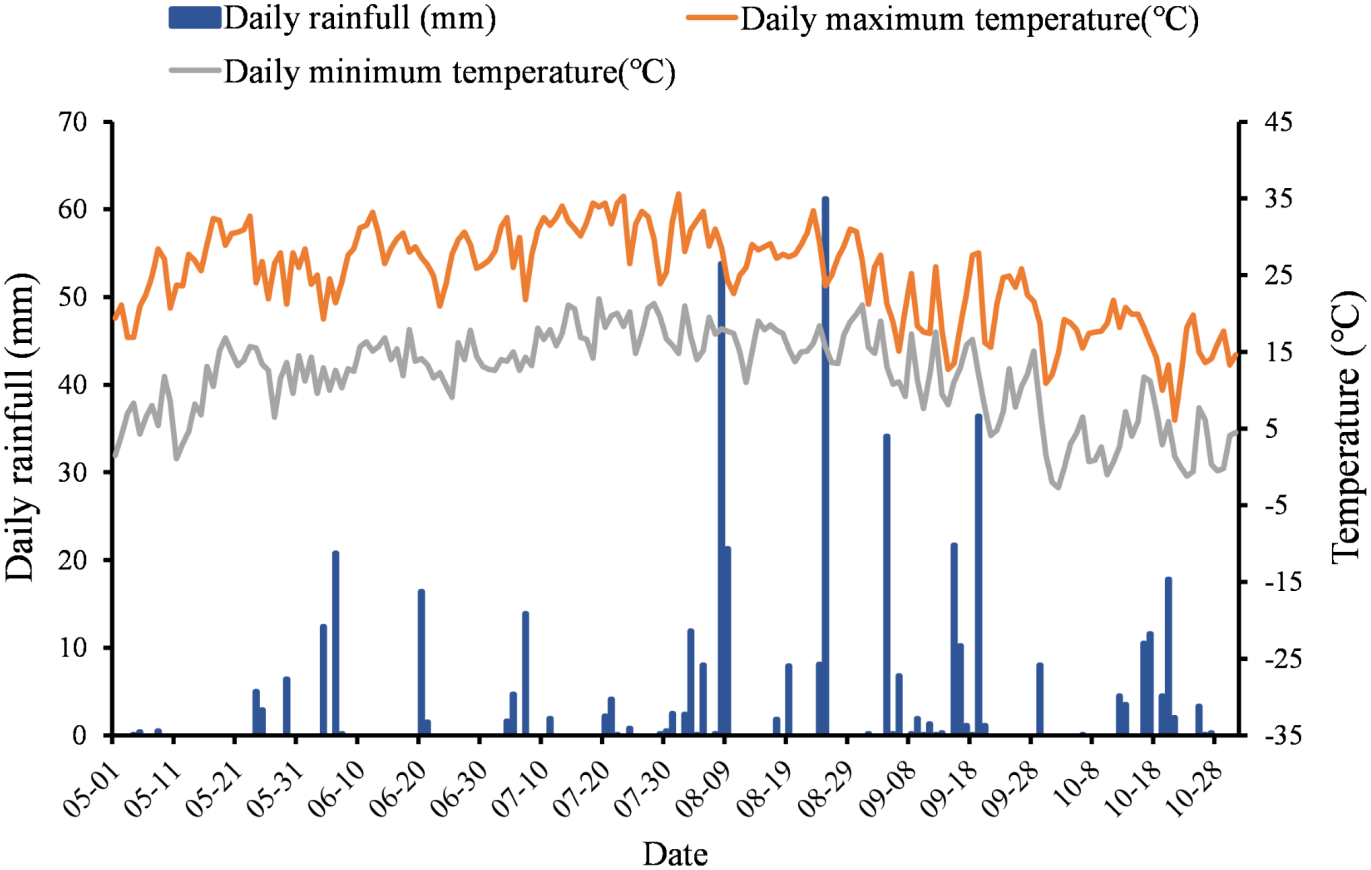
Main meteorological factors during the growing period in the experimental area.

The experiment was conducted from May to October 2024 in the Inner Mongolia Autonomous Region of the People’s Republic of China Ordos Etuoke Banner Agricultural and Animal Husbandry Technology Extension Station (39°06′ N, 107°30′ E, altitude 1151 m), **Figure 1**. The region is characterized by a typical temperate semi-arid continental climate, with a mean annual temperature of 6.5-8.5 °C and annual precipitation of approximately 250 mm, most of which occurs from July to September. Annual sunshine duration exceeds 3,000 h, with large diurnal temperature fluctuations and a frost-free period of approximately 122 days. The experimental soil is classified as sandy loam, with good aeration but limited ability to retain water and nutrients. Before the experiment, the 0–20 cm soil layer contained 8.01 g kg^-^¹ organic matter, 14 mg kg^-^¹ alkali-hydrolyzable nitrogen, 0.41 mg kg^-^¹ available phosphorus, 125 mg kg^-^¹ available potassium, and had a pH of 9.4. The primary meteorological parameters, including maximum and minimum air temperatures as well as precipitation, were recorded in real time by the automatic weather station located in the test area (**Figure 2**).

### 2.2 Experimental design and management

The maize variety utilized was “Guorui 188,” which was sown in May 2024 and harvested in October 2024. The experiment was arranged in a randomized complete block design with two factors: deficit irrigation level and straw mulching. Six treatment combinations were established, including three deficit irrigation levels: light (W1: 2625 m³ ha^-^¹), moderate (W2: 2400 m³ ha^-^¹), and severe (W3: 2175 m³ ha^-^¹). These deficit irrigation levels were determined according to the local water use quota standard of the Inner Mongolia Autonomous Region (DB15/T385-2020) (Market Supervision and Administration of Inner Mongolia Autonomous Region: Inner Mongolia Autonomous Region, 2020). The deficit irrigation amounts under W1, W2, and W3 corresponded to 80%, 73%, and 66% of the local conventional irrigation amount of 3300 m³ ha^-^¹, respectively. Two straw mulching levels were established: F1 (9000 kg ha^-^¹) and F2 (0 kg ha^-^¹). Each treatment was replicated three times.

During the whole maize growing period, irrigation was conducted 12 times. Irrigation was controlled by dynamic monitoring of field soil moisture content and was initiated when soil moisture content decreased below the preset threshold. The threshold was defined with reference to field capacity and adjusted according to maize growth stage and deficit irrigation level, thereby establishing a gradually decreasing soil moisture control gradient from W1 to W3. The distribution of irrigation among growth stages was based on field soil moisture monitoring and the stage-specific water demand of maize, particularly the high water requirement during the tasseling-silking and grain-filling stages (Sah et al., 2020). The numbers of irrigation events at the emergence, seedling, jointing, tasseling-silking, grain-filling, and maturity stages were 1, 1, 2, 4, 3, and 1, respectively. The irrigation quota for each irrigation event was 218.75, 200.00, and 181.25 m³ ha^-^¹ for W1, W2, and W3, respectively.

**Figure 3:**
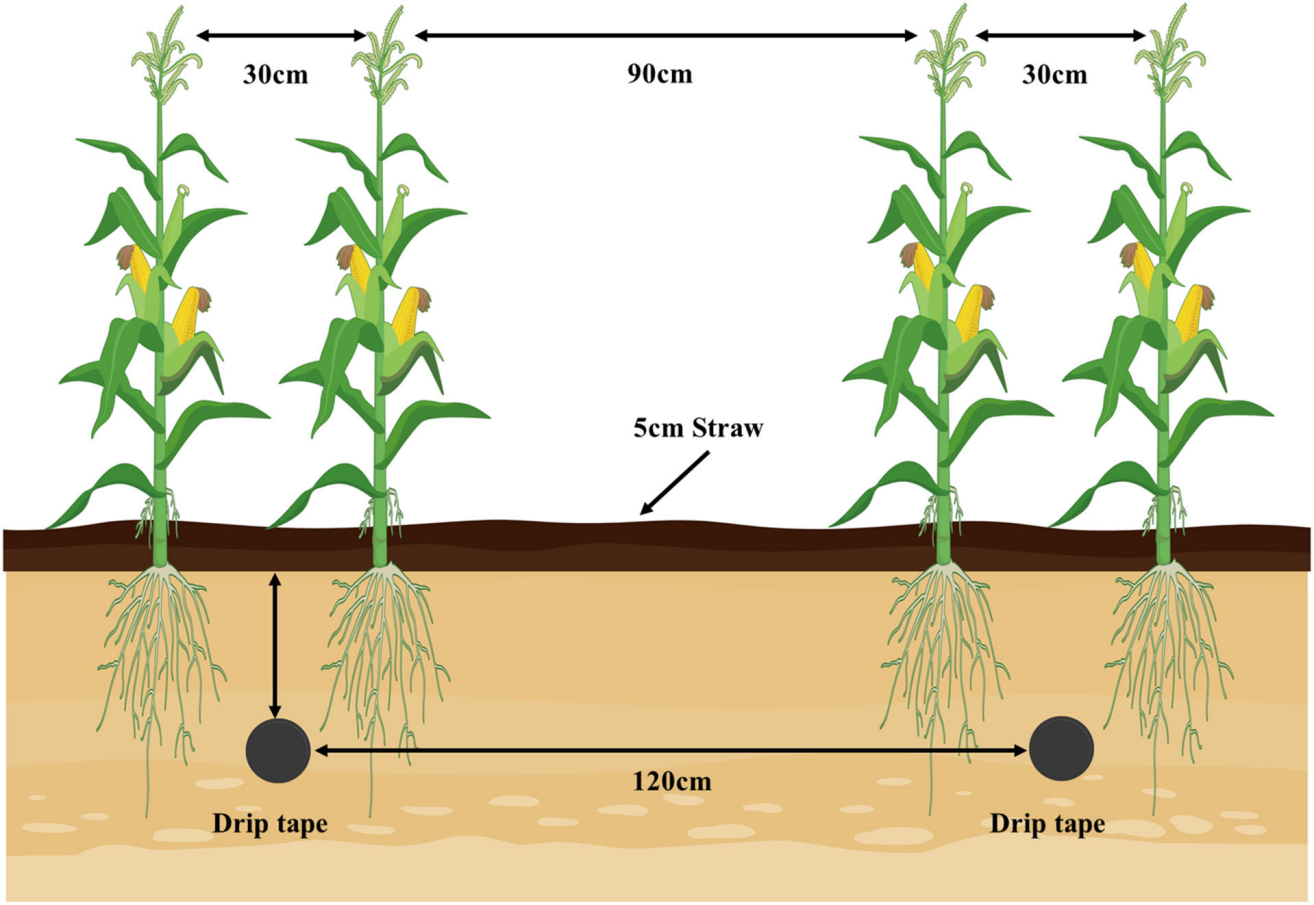
Layout of the field experiment.

After mechanical crushing, the straw was returned to the field, with the particle size maintained between 4 and 6 cm. The crushed straw was evenly distributed between wide and narrow rows, achieving a covering thickness of approximately 5 cm. Planting was conducted in alternating wide and narrow rows, with drip irrigation tape and double-row sowing arranged between the narrow rows. The spacing was 90 cm for wide rows, 30 cm for narrow rows, and 20 cm between plants. Each plot measured 24 × 46 m (**Figure 3**).

Irrigation water was obtained from the groundwater wells in the high-standard farmland demonstration area. Each plot was equipped with independent water delivery pipelines and water meters, facilitating precise irrigation. The irrigation system used underground drip irrigation, utilizing specialized shallow-buried drip irrigation pipes with resistance to negative pressure and rodent damage, developed by Dayu Water Conservation Group. The drip irrigation tape was installed at a depth of 25 cm, with emitters spaced 30 cm apart and drip irrigation tapes positioned 120 cm apart. The emitters were designed to deliver a flow rate of 2 L h^-^¹. Installation was conducted using a rotary tillage, ridging, and drip irrigation tape integrated machine. During the irrigation process, an integrated approach to water and fertilizer management was employed. Fertilization strategies were informed by local maize production practices, ensuring consistency across treatment plans. Field management practices, including weeding and pest and disease control, were uniformly executed in accordance with the local conventional agricultural production model.

### 2.3 Sampling, determination and calculation methods

#### 2.3.1 Soil sample collection

Soil samples were collected at the maturity stage of maize in October 2024. A five-point sampling method (Yang et al., 2008) was employed to obtain soil samples from the 0–20 cm layer using a 70 mm diameter soil auger, targeting narrow rows within each plot. The samples from the five points were thoroughly mixed and passed through a 2 mm sieve to eliminate plant roots, animal remains, and other impurities. The samples were naturally dried in a cool, ventilated area, and stored under light protection.

#### 2.3.2 Determination of soil nutrients

For total nutrient determination, the soil samples were finely ground to ensure complete digestion or fusion. For total nitrogen determination by Kjeldahl method, samples that had passed through a 2 mm sieve were further ground to pass through a 0.25 mm sieve, followed by Kjeldahl digestion, distillation, and titration (Bremner, 1965). For total phosphorus determination, a subsample was further ground to pass through a 0.149 mm sieve, followed by alkali fusion and determination using the Mo-Sb anti spectrophotometric method (Guo et al., 2024). For total potassium determination, the soil sample was ground to pass through a 0.15 mm sieve. Approximately 0.25 g of the prepared sample was fused with NaOH to prepare the test solution, and potassium was then determined by flame photometry (Wan et al., 2025).

For available nutrient determination, air-dried soil samples with the required sieve size were used for diffusion or extraction. For alkaline hydrolysable nitrogen determination by alkali hydrolysis diffusion method, 2.00 g of air-dried soil passed through a 1 mm sieve was mixed with ferrous sulfate and 1 mol L^-^¹ NaOH. The sample was incubated for alkaline hydrolysis diffusion at 38 ± 2 °C for 24 ± 0.5 h. The released NH_3_ was absorbed by boric acid indicator solution and titrated with 0.005 or 0.01 mol L^-^¹ standard sulfuric acid solution. For available phosphorus determination, 2.50 g of soil sample was extracted with 50.0 mL of 0.5 mol L^-^¹ NaHCO_3_ solution at 25 ± 1 °C by shaking at 180–200 r min^-^¹ for 30 ± 1 min. The filtrate was then determined using the Mo-Sb anti spectrophotometric method (Yu et al., 2025). For available potassium determination, 2.00 g of air-dried soil passed through a 1 mm sieve was extracted with 20 mL of 1 mol L^-^¹ neutral NH_4_OAc solution by shaking at 220 rpm for 30 min. The filtrate was then determined by flame photometry (Yu et al., 2025).

#### 2.3.3 Determination of soil organic carbon fractions

Total organic carbon was determined using the potassium dichromate volumetric method. Light fraction organic carbon was measured using the density fractionation method with a sodium tungstate solution of 1.85 g cm^-^³ density (Gelman et al., 2012). Easily oxidizable organic carbon was determined using the potassium permanganate oxidation method (Blair et al., 1995). Fine particulate organic carbon and coarse particulate organic carbon were determined using the wet sieving method with a particle size boundary of 53 µm (Blair et al., 1995).

#### 2.3.4 Determination of soil enzyme activity

Urease activity was determined using the sodium phenolate-sodium hypochlorite colorimetric method after incubation with urea solution at 37 °C for 24 h, and the released NH_3_-N was quantified at 578 nm using an NH_3_-N standard curve. Catalase activity was determined by potassium permanganate titration based on the residual H_2_O_2_ after enzymatic decomposition. Phosphatase activity was determined using the disodium phenyl phosphate colorimetric method after incubation with disodium phenyl phosphate substrate at 37 °C for 24 h, and the released phenol was measured at 660 nm using a phenol standard curve. Sucrase and cellulase activities were determined using the 3,5-dinitrosalicylic acid colorimetric method, and the released reducing sugars were measured at 508 nm using a glucose standard curve. Protease activity was determined using the ninhydrin colorimetric method after incubation with casein substrate at 30 °C for 24 h, and the released amino nitrogen was measured at 500 nm using a glycine standard curve (Guan,S.Y., 1986). Detailed sample weights, assay mixtures, extraction or filtration steps, calibration curves, blank controls, and calculation formulas are provided in the Supplementary Material.

#### 2.3.5 Determination of yield

At maize maturity, a 3 m² harvest area was designated in each plot, and ear weight, ear length, grain number per ear, and 100 grain weight were measured directly in the field to calculate theoretical yield. Each treatment was replicated three times.

#### 2.3.6 Calculation of water use efficiency and irrigation water use efficiency

Water use efficiency (WUE, kg m^-^³) was calculated as follows:


$$WUE=\frac{Y}{ET}$$

Y is grain yield (kg ha^-^¹), ET is evapotranspiration (mm).


ET=P+I+ΔSWS−D−R

P is precipitation (mm), I is irrigation amount (mm), ΔSWS is the change in soil water storage in the measured soil layer between planting and maturity (mm), D is deep percolation (mm) and R is surface runoff (mm) (Gu et al., 2023).

Irrigation water use efficiency (IWUE, kg m^-^³) was calculated as follows:


$$IWUE=\frac{Y}{I}$$

Y is grain yield (kg ha^-^¹), I is irrigation amount (mm) (Ouyang et al., 2025).

#### 2.3.7 Calculation of soil quality index

The soil quality index (SQI) was used to evaluate the effects of deficit irrigation and straw mulching on soil quality. In this study, SQI was established using the total data set method based on 17 soil factors. All 17 soil indicators used for SQI construction were regarded as positive soil quality indicators and were therefore standardized using the “more is better” scoring function. The calculation procedure was as follows. First, all soil factors were converted into scores ranging from 0 to 1 using the “more is better” scoring function and the “less is better” scoring function:


$$S_{i}^{'}=\frac{x_{i}-x_{min}}{x_{max}-x_{min}}$$

$$S_{i}^{''}=\frac{x_{max}-x_{i}}{x_{max}-x_{min}}$$

$S_{i}^{'}$ and​ $S_{i}^{''}$are the scores of the i-th soil factor, and $x_{i}$, $x_{max},and\;x_{min}\;$ are the measured value, maximum value, and minimum value of the i-th soil factor, respectively. Then, the weight of each soil factor ( $W_{i}$) was calculated using principal component analysis (PCA) according to the ratio of the communality of each factor to the total communality of all factors (Chang et al., 2023).

Soil quality index (SQI) was calculated as follows:


$$SQI=\sum_{i=1}^{n}W_{i}\times S_{i}$$

n is the number of factors, and $W_{i}$​ and $S_{i}$​ are the weight and score of each factor, respectively (Chen et al., 2021).

### 2.4 Statistical analyses

All statistical analyses were performed using SPSS 27 (IBM Corp., Armonk, NY, USA). Principal component analysis (PCA) was conducted using OriginPro 2021 (OriginLab Corporation, Northampton, MA, USA). Structural equation modeling (SEM) was performed using AMOS 28 (IBM Corp., Armonk, NY, USA). Given the limited sample size, principal component analysis (PCA) was applied prior to SEM construction to reduce multicollinearity among variables and simplify the model structure. Before PCA, the suitability of the data for dimensionality reduction was evaluated using the Kaiser–Meyer–Olkin (KMO) test and Bartlett’s test of sphericity. PCA was performed only when the KMO > 0.5 and Bartlett’s test was significant (*P* < 0.05), indicating that the variables were suitable for factor extraction (Bartlett, 1954; Kaiser, 1974). Soil nutrient, enzyme activity and soil organic carbon fractions indicators were subjected to PCA, and the first principal component (PC1) was extracted as a composite variable. PC1 explained the largest proportion of variance and was therefore used to represent the overall variation in soil biochemical properties. This composite variable, together with yield-related indicators, was subsequently incorporated into the SEM model (Chen et al., 2013; Abdel-Fattah et al., 2021; Aishwarya et al., 2025). Model fit was evaluated based on the following criteria: χ²/DF < 3, GFI > 0.90, CFI > 0.90, RMSEA < 0.08, and *P* > 0.05. Figures were generated using OriginPro 2021 and GraphPad Prism 10.6.

## 3 Results

### 3.1 Soil nutrients and enzyme activities

**Figure 4:**
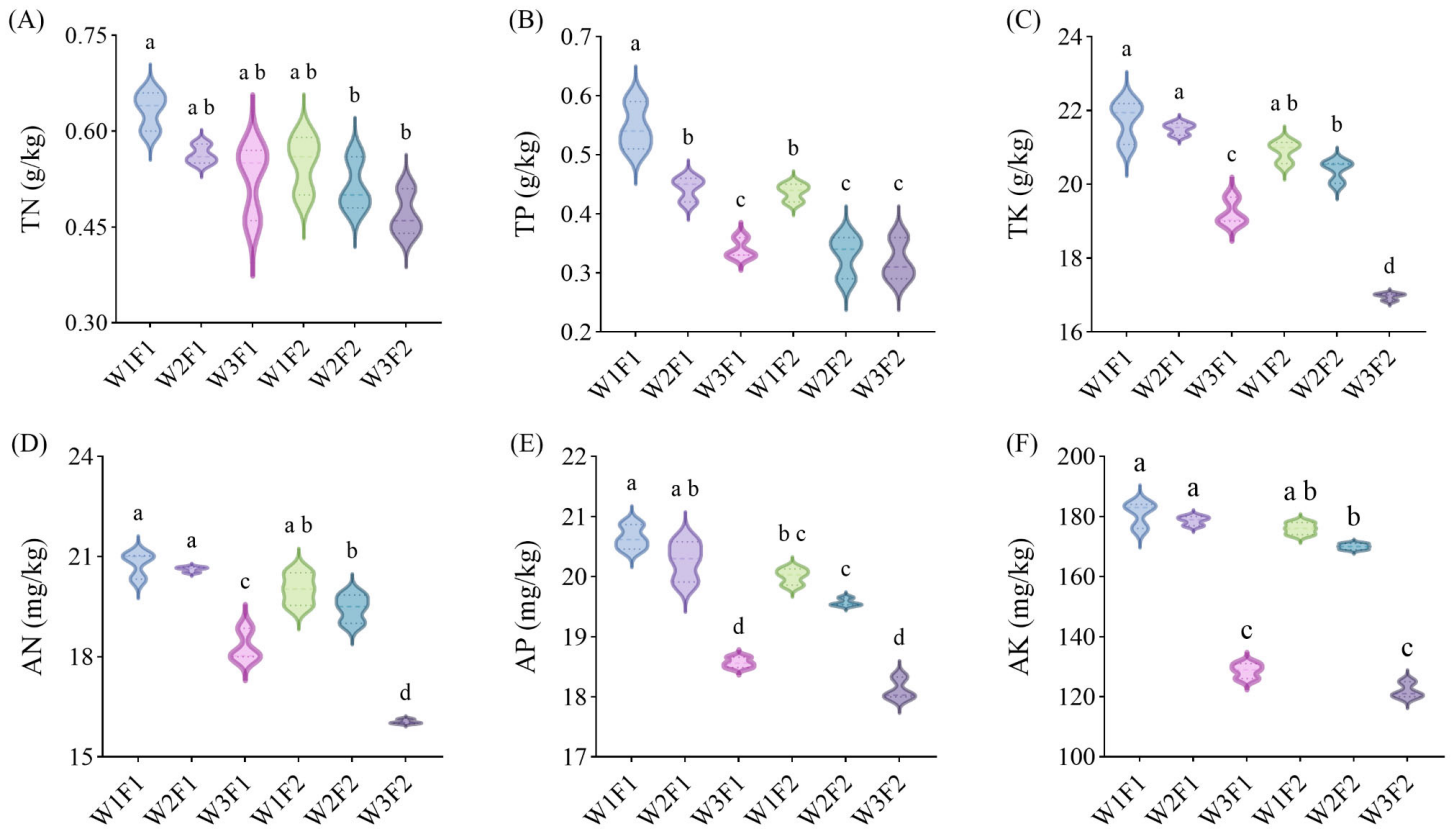
Effects of deficit irrigation and straw mulching on soil nutrient contents. **(A)** Total nitrogen (TN), **(B)** total phosphorus (TP), **(C)** total potassium (TK), **(D)** alkaline hydrolysable nitrogen (AN), **(E)** available phosphorus (AP), and **(F)** available potassium (AK). Different letters indicate significant differences among treatments (*P* < 0.05).

Soil nutrient contents varied significantly among deficit irrigation and straw mulching treatments (**Figure 4**). Under the same deficit irrigation level, straw mulching (F1) consistently increased total nitrogen (TN; **Figure 4A**), total phosphorus (TP; **Figure 4B**), and total potassium (TK; **Figure 4C**) compared with no mulching (F2). TN increased by 9.7-15.2%, TP by 6.3-34.3%, and TK by 4.0-13.4% under light (W1), moderate (W2), and severe (W3) deficit irrigation. Among the available nutrients, alkali-hydrolyzable nitrogen (AN; **Figure 4D**) and available potassium (AK; **Figure 4F**) showed the greatest increases under W3, rising by 14.0% and 5.5% under F1 relative to F2. Under the same mulching treatment, soil nutrient contents generally declined as deficit irrigation intensified. Under F1, TN reached 0.633 g kg^-^¹ in W1 but decreased under W3; similarly, TP and AK were 60.8% and 40.7% higher in W1 than in W3. The highest soil nutrient contents were observed under W1F1, with TN, TP, and AK reaching 0.633 g kg^-^¹, 0.547 g kg^-^¹, and 181 mg kg^-^¹, respectively, which were higher than those under W3F2.

**Figure 5:**
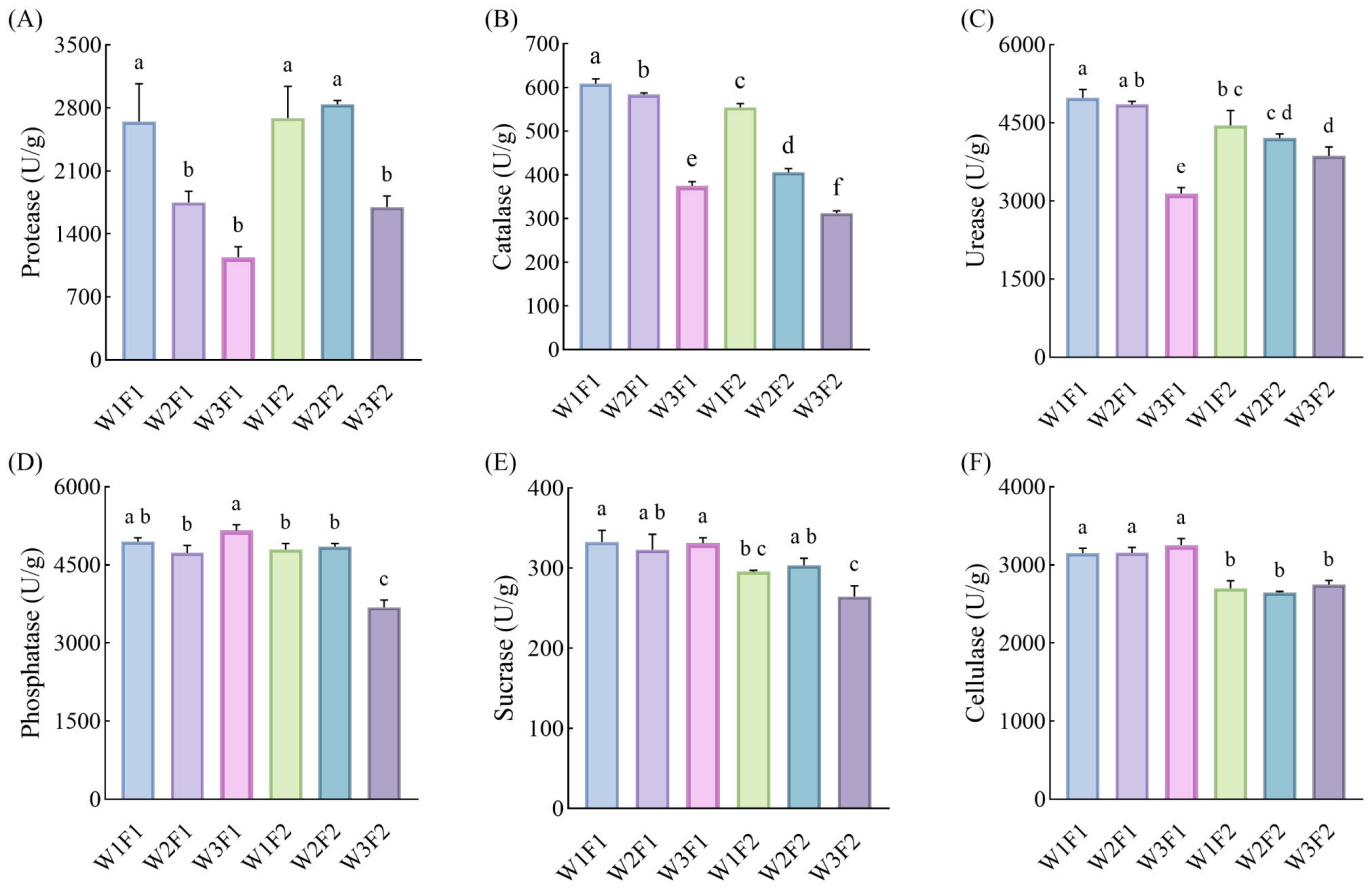
Effects of deficit irrigation and straw mulching on soil enzyme activities. **(A)** Protease, **(B)** Catalase, **(C)** Urease, **(D)** Phosphatase, **(E)** Sucrase, and **(F)** Cellulase. Different letters indicate significant differences among treatments (*P* < 0.05).

Soil enzyme activities also varied significantly among treatments (**Figure 5**). Protease (**Figure 5A**), catalase (**Figure 5B**), and urease (**Figure 5C**) activities were generally higher under W1 and W2 than under W3. Protease activity under F1 was 132.0% and 53.3% in W1 and W2 higher than in W3, respectively, while under F2 it was 58.0% and 67.2% higher, respectively. Catalase activity under F1 increased by 62.2% and 55.5% in W1 and W2 relative to W3, respectively, and under F2 increased by 77.6% and 30.1%, respectively. Urease activity under F1 was 58.6% and 54.4% higher in W1 and W2 than in W3, respectively, while under F2 it was 15.2% and 8.9% higher, respectively. Phosphatase (**Figure 5D**), sucrase (**Figure 5E**), and cellulase (**Figure 5F**) activities were more strongly influenced by straw mulching. Under W3, phosphatase activity under F1 was 39.9% higher than under F2. Sucrase activity under F1 exceeded that under F2 at the same deficit irrigation levels. Although cellulase activity was generally significantly higher under F1 than under F2, no significant differences were observed among irrigation treatments within the same straw mulching level. The highest catalase, urease, and sucrase activities were recorded under W1F1 (**Figures 5B, C, E**), reaching 608.61, 4981.94, and 332.46 U g^-^¹. In contrast, phosphatase and cellulase peaked under W3F1 (**Figures 5D, F**), where phosphatase reached 5161.57 U g^-^¹ and cellulase reached 3255.73 U g^-^¹. Their lowest values were observed under W3F2, at 3689.28 U g^-^¹ for phosphatase and 2747.33 U g^-^¹ for cellulase.

### 3.2 Soil organic carbon fractions and their proportions

**Figure 6:**
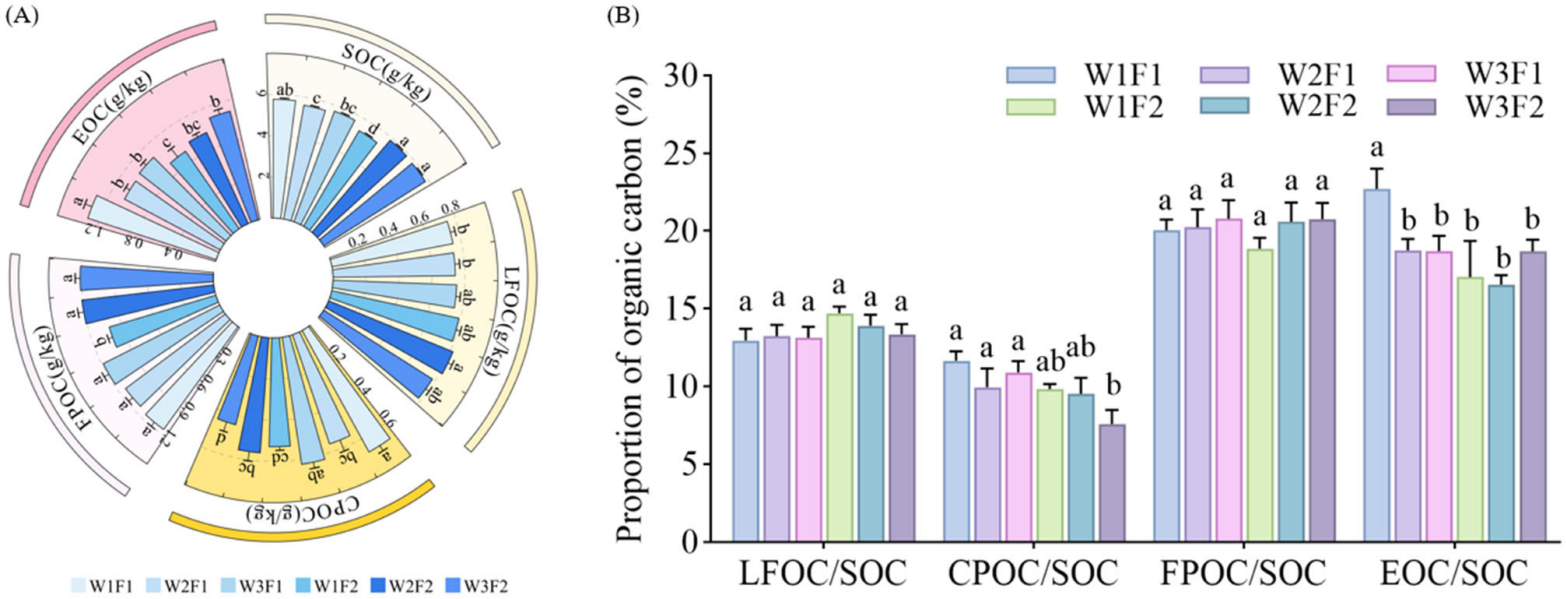
Effects of deficit irrigation and straw mulching on soil organic carbon fractions. **(A)** Contents of soil organic carbon fractions. **(B)** Proportions of soil organic carbon fractions relative to total soil organic carbon. Different letters indicate significant differences among treatments (*P* < 0.05).

Soil organic carbon fractions differed significantly among deficit irrigation and straw mulching treatments (**Figure 6A**). Soil organic carbon (SOC) was 5.869 g kg^-^¹ under W2F2 and 5.841 g kg^-^¹ under W3F2, while fine particulate organic carbon (FPOC) reached 1.208 and 1.213 g kg^-^¹ under the same treatments. Under the same deficit irrigation level, light fraction organic carbon (LFOC) was generally higher under F2 than under F1. Compared with W1F1, LFOC under W1F2, W2F2, and W3F2 increased by 5.8%, 9.2%, and 4.6%. Under W1F1, coarse particulate organic carbon (CPOC) reached 0.673 g kg^-^¹, whereas easily oxidizable organic carbon (EOC) reached 1.310 g kg^-^¹. Under F1, CPOC in W2F1 was lower than in W1F1, decreasing by 16.8%. Under F2, CPOC declined with increasing irrigation deficit, with W3F2 decreasing by 15.9% compared with W1F2. For EOC under F1, W2F1 decreased by 19.6% and W3F1 decreased by 18.4% compared with W1F1. Under F2, EOC reached its lowest value in W1F2 (0.915 g kg^-^¹), which was 30.1% lower than that in W1F1.

The proportions of different soil organic carbon fractions relative to SOC also varied significantly among treatments (**Figure 6B**). FPOC accounted for the largest proportion (18.87-20.79%), followed by EOC (16.57-22.71%). CPOC represented 7.60-11.67% of SOC, whereas LFOC accounted for the smallest proportion (12.96-14.70%). Under different treatments, LFOC proportions increased by 1.75% in W1F2 and by 0.43% in W3F2. In contrast, CPOC proportions decreased by 1.72% in W2F2 and by 4.14% in W3F2. FPOC proportions showed relatively small variations across treatments. EOC proportions were highest under W1F1 (22.71%) and W3F2 (18.68%), whereas W2F2 showed a significant decrease to 16.57%.

### 3.3 Integrated soil quality assessment based on SQI

**Figure 7:**
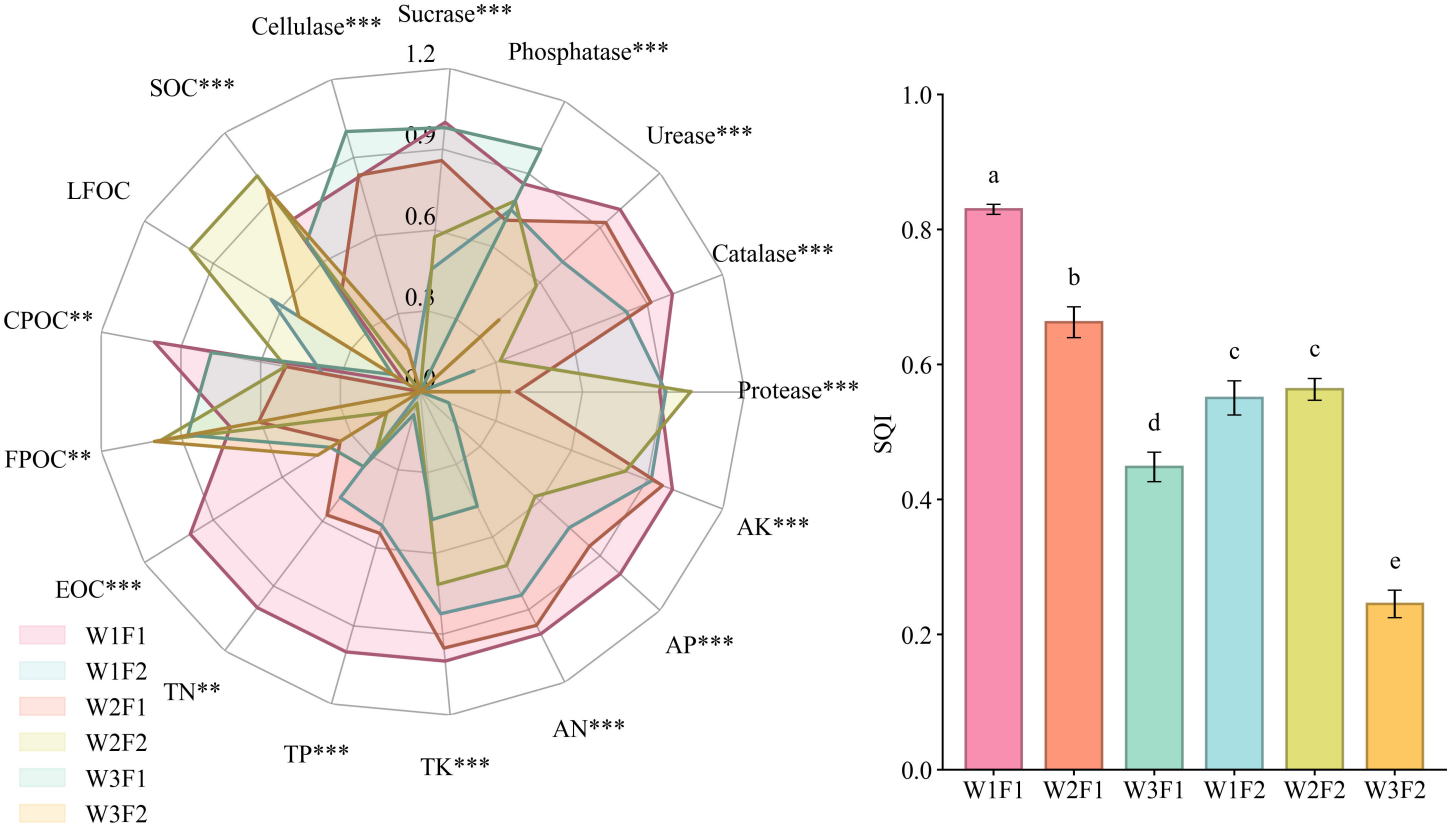
Relative responses of soil quality indicators and soil quality index under different irrigation levels and straw mulching treatments. **(A)** The radar plot shows the relative responses of Cellulase; Sucrase; Phosphatase; Urease; Catalase; Protease; TN, total nitrogen; TP, total phosphorus; TK, total potassium; AN, alkaline hydrolysable nitrogen; AP, available phosphorus; AK, available potassium; SOC, soil organic carbon; LFOC, light fraction organic carbon; CPOC, coarse particulate organic carbon; FPOC, fine particulate organic carbon; EOC, easily oxidizable organic carbon. **(B)** Soil quality index under different treatments. Different letters indicate significant differences among treatments (*P* < 0.05). ** and *** indicate significance at P < 0.01 and P < 0.001, respectively.

Significant differences in SQI were observed among the deficit irrigation and straw mulching treatments (**Figure 7B**). The highest SQI was observed under W1F1, reaching 0.8296, followed by W2F1, with a value of 0.6625, whereas the lowest SQI was observed under W3F2, with a value of 0.2451. Under the same deficit irrigation level, straw mulching increased SQI. Specifically, under W1, W2, and W3 conditions, straw mulching increased SQI by 50.7%, 17.6%, and 82.8%, respectively. W1F1 and W2F1 both showed relatively high SQI values, indicating that straw mulching combined with light or moderate deficit irrigation was more favorable for maintaining integrated soil quality.

The standardized scores of individual soil indicators further explained the differences in SQI among treatments (**Figure 7A**). W1F1 had high scores for most nutrient indicators, including TN, TP, TK, AN, AP, and AK, and also showed high scores for catalase, urease, sucrase, SOC, CPOC, and EOC. These high scores were consistent with the highest SQI observed under W1F1. W2F1 also maintained high scores for catalase, urease, sucrase, cellulase, TK, AN, AP, and AK, and therefore still showed a relatively high SQI under moderate deficit irrigation. In contrast, W3F2 had low scores for most nutrient indicators, several enzyme activities, and CPOC, resulting in the lowest SQI. These results indicate that differences in SQI among treatments were mainly associated with coordinated changes in soil nutrients, enzyme activities, and some labile organic carbon fractions.

### 3.4 Maize yield and its relationship with soil quality

Deficit irrigation and straw mulching significantly affected maize yield, ear number, grain number per ear, 1000-grain weight, WUE, and IWUE, with significant interaction effects between the two factors on these indicators (P < 0.05; **Table 1**). The yield under W2F1 did not differ significantly from that under W1F1 but was significantly higher than that under the other four treatments (*P* < 0.05). W2F1 produced the greatest ear number and grain per ear, reaching 59.71 × 10³ ha^-^¹ and 538.12 kernels, respectively. The highest 1000-grain weight was observed under W1F1 (247.48 g). Compared with W1F1, grain yield decreased by 9.4% in W1F2, 0.6% in W2F1, 9.8% in W2F2, 28.8% in W3F1, and 34.9% in W3F2. Both W1F1 and W2F1 showed clear yield advantages. When the irrigation deficit intensified to W3, yield declined significantly, and the yield-promoting effect of straw mulching weakened. In terms of water use efficiency, W2F1 showed the highest WUE and IWUE among all treatments, reaching 11.71 and 6.88 kg m^-^³, respectively. Compared with W1F1, W2F1 maintained a similar grain yield while reducing the seasonal irrigation amount, increasing WUE and IWUE by 8.0% and 10.8%, respectively. Under the same deficit irrigation level, WUE and IWUE were higher under straw mulching than under no mulching. In contrast, W3F2 showed the lowest WUE and IWUE, with values of 6.85 and 4.17 kg m^-^³, respectively. These results indicate that moderate deficit irrigation combined with straw mulching improved water productivity while maintaining maize yield, whereas severe deficit irrigation reduced both yield and water-use performance.

**Table 1:** Effects of different treatments on maize yield, ear number, grain number per ear, 1000-grain weight, WUE, and IWUE.

Treatment	Yield/kg ha^-1^	Ear number/10^3^ ha^-1^	Grain number per ear	1000-grain weight/g	WUE/kg m^-3^	IWUE/kg m^-3^
W1F1	6642.54 ± 57.12 a	59.12 ± 0.43 a	534.12 ± 9.12 a	247.48 ± 2.91 a	10.84 ± 0.09b	6.21 ± 0.05b
W2F1	6602.38 ± 96.87 a	59.71 ± 0.91 a	538.12 ± 6.05 a	241.74 ± 4.28 a	11.71 ± 0.17a	6.88 ± 0.10a
W3F1	4728.54 ± 71.29 c	53.28 ± 0.85 c	538.91 ± 7.91 a	193.74 ± 2.11 c	9.86 ± 0.15c	5.64 ± 0.09c
W1F2	6018.54 ± 98.14 b	57.09 ± 1.28 b	541.66 ± 4.61 a	229.97 ± 4.87 b	8.74 ± 0.14d	5.01 ± 0.08d
W2F2	5987.88 ± 89.64 b	55.67 ± 1.56 b	540.88 ± 8.95 a	233.88 ± 1.91 b	7.59 ± 0.11e	4.62 ± 0.07e
W3F2	4321.71 ± 62.19 d	54.12 ± 2.01 c	529.24 ± 1.98 b	177.51 ± 1.86 d	6.85 ± 0.10f	4.17 ± 0.06f
W	**	*	**	*	***	***
F	*	**	**	**	***	***
W×F	***	**	***	**	***	***

Values represent mean ± SE (n = 3). Different letters indicate significant differences among treatments (*P* < 0.05). *, **, and *** indicate significance at *P* < 0.05, *P* < 0.01, and *P* < 0.001, respectively.

**Figure 8:**
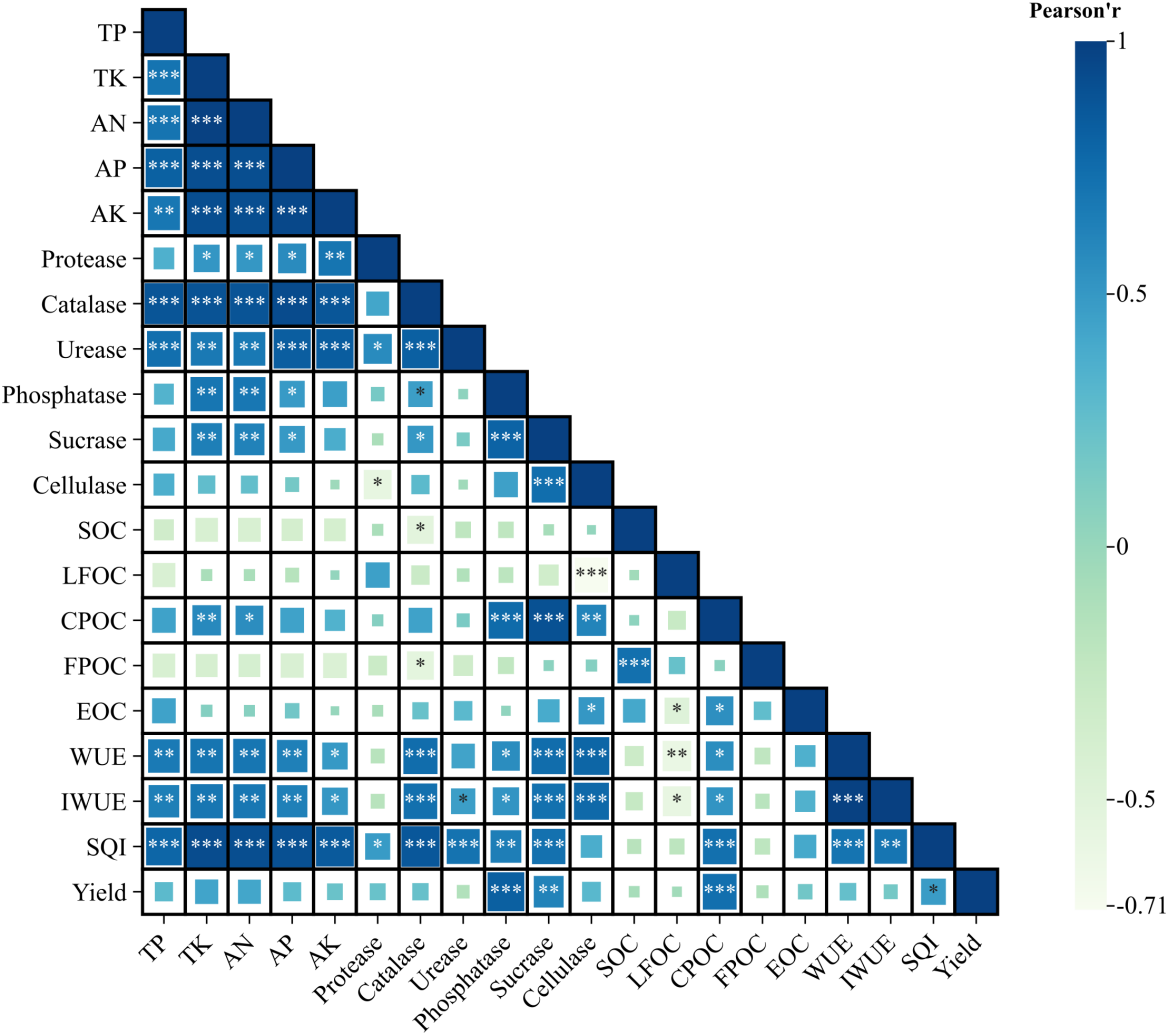
Pearson correlation analysis among soil nutrients, organic carbon fractions, enzyme activities, maize yield and SQI. Colors represent the strength and direction of Pearson correlation coefficients. *, **, and *** indicate significance at *P* < 0.05, *P* < 0.01, and *P* < 0.001, respectively. TN, total nitrogen; TP, total phosphorus; TK, total potassium; AN, alkaline hydrolysable nitrogen; AP, available phosphorus; AK, available potassium; SOC, soil organic carbon; LFOC, light fraction organic carbon; CPOC, coarse particulate organic carbon; FPOC, fine particulate organic carbon; EOC, easily oxidizable organic carbon.

Pearson correlation analysis showed significant relationships among soil quality indicators, SQI, maize yield, WUE, and IWUE (**Figure 8**). Maize yield was significantly and positively correlated with phosphatase, CPOC, sucrase, and SQI, with correlation coefficients of 0.827, 0.741, 0.628, and 0.481, respectively. SQI was significantly and positively correlated with maize yield, WUE, and IWUE, as well as with several nutrient indicators and enzyme activities, including TK, AN, AP, catalase, AK, TP, CPOC, sucrase, and urease. WUE and IWUE were highly positively correlated with each other and were also significantly correlated with SQI, cellulase, sucrase, catalase, and several nutrient indicators. Maize yield, WUE, IWUE, and SQI showed significant correlations with several soil nutrient, enzyme activity, and organic carbon fraction indicators.

**Figure 9:**
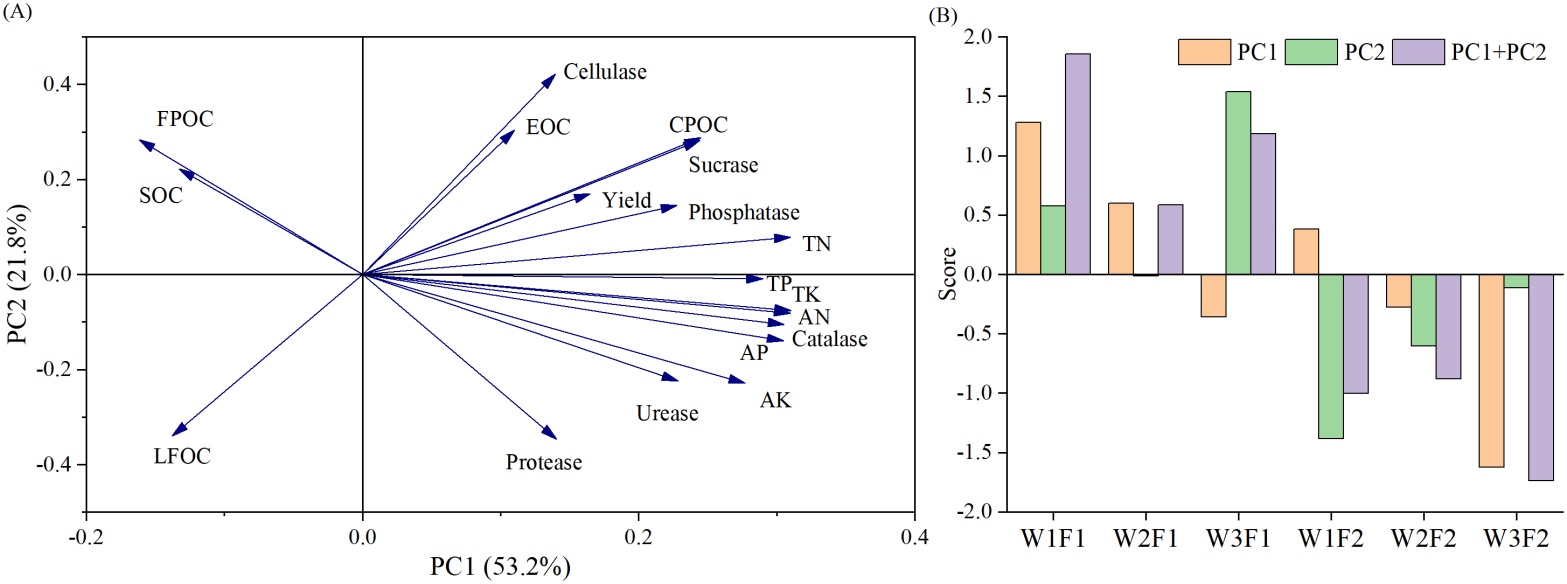
Principal component analysis (PCA) of soil nutrients, organic carbon fractions, enzyme activities, and maize yield. **(A)** PCA biplot showing the relationships among soil nutrients, organic carbon fractions, enzyme activities, and maize yield. **(B)** Scores of different treatments on the principal components. PC1 and PC2 explain 53.2% and 21.8% of the total variance, respectively.

Principal component analysis indicated that the first two principal components explained 75.0% of the total variance (**Figure 9A**). PC1 accounted for 53.2% of the variance, while PC2 explained 21.8%. The distribution of variables in the ordination space was primarily associated with PC1. Protease, sucrase, cellulase, CPOC, and maize yield had high loadings on PC1, whereas LFOC was distributed in the opposite direction. SOC and FPOC were mainly associated with PC2. In terms of treatment scores (**Figure 9B**), W1F1 and W3F1 showed relatively high composite scores, clearly exceeding those of other treatments. Treatments without straw mulching (F2) generally had lower scores, with W3F2 ranking lowest.

Structural equation modeling further clarified the pathways through which deficit irrigation and straw mulching affected maize yield via soil nutrients, enzyme activity, and soil organic carbon fractions. The model showed a good fit, with χ²/DF = 0.453, CFI = 1.000, GFI = 0.969, RMSEA = 0.00, and *P* = 0.770, and explained 72.6% of the variation in maize yield. Deficit irrigation significantly affected soil nutrients, enzyme activity, and soil organic carbon fractions, whereas straw mulching significantly affected soil nutrients and enzyme activity but had no significant direct effect on soil organic carbon fractions. Enzyme activity had a significant positive direct effect on maize yield, while soil organic carbon fractions showed positive effects. Deficit irrigation had a significant negative direct effect on yield, whereas straw mulching had a significant positive direct effect on yield. These results suggest that maize yield responses under deficit irrigation and straw mulching were mainly associated with the direct effects of water and mulching management and with changes in enzyme activity and organic carbon fractions.

## 4 Discussion

### 4.1 Effects of deficit irrigation and straw mulching on soil nutrients and enzyme activities

The present study demonstrated that deficit irrigation and straw mulching affected soil nutrient contents and enzyme activities under different deficit irrigation levels. With increasing irrigation deficit intensity, TN, TP, AN, and AK generally declined, with the greatest reductions observed under W3 (**Figure 4**). These findings are consistent with Zhang et al. (2024), who found that reduced irrigation decreased soil nitrogen and phosphorus contents in maize fields in semi-arid environments. Under the same irrigation deficit level, soil nutrient contents were consistently higher under F1 than under F2. Straw mulching mitigated nutrient depletion and helped maintain soil fertility status. This result is in agreement with previous studies by Ning et al. (2024) and Liu et al. (2024), which showed that mulching practices improve soil nutrient availability and enzyme activities under limited irrigation conditions.

Soil enzyme activities further reflected the above changes in nutrient status (**Figure 5**). Urease and protease, which are closely associated with nitrogen transformation processes (Piotrowska-Długosz and Wilczewski, 2014), together with catalase, an indicator of soil redox status (Gu et al., 2019), were higher under W1 and W2 than under W3. This suggests that these enzymes are sensitive to increasing irrigation deficit intensity (Jia et al., 2018). In contrast, phosphatase, sucrase, and cellulase showed more pronounced increases under straw mulching, particularly under W3. For sucrase and cellulase, no significant differences were observed among deficit irrigation treatments within the same straw mulching level (**Figures 5E, F**). This indicates that sucrase and cellulase activities responded weakly to the deficit irrigation gradient in this study. Sucrase is involved in sucrose hydrolysis (Wang et al., 2020), while cellulase catalyzes cellulose degradation (Sharma et al., 2024) and plays an important role in crop residue decomposition in soil. These enzyme activities may be closely related to straw-derived substrate supply and soil microbial processes. Akhtar et al. (2018) reported that straw mulching enhanced soil enzyme activity by improving substrate supply and soil hydrothermal conditions. Ning et al. (2024) also showed that soil enzyme activity responses to irrigation can vary with irrigation regime and microbial processes. The irrigation gradient may not have been strong enough to cause clear differences in sucrase and cellulase activities within the same straw mulching level. Soni et al. (2021) have shown that mulching practices can maintain relatively high enzyme activity levels under deficit irrigation conditions. These findings further support previous reports that similar trends occur across different irrigation deficit levels. In conclusion, under semi-arid conditions, straw mulching mitigated the negative effects of deficit irrigation on soil nutrient supply. This effect was associated with higher soil nutrient contents and increased activities of key enzymes, including urease, phosphatase, sucrase, and cellulase. Among the treatments, W1F1 exhibited relatively superior performance in several nutrient indicators and key enzyme activities, suggesting that moderate deficit irrigation combined with straw mulching is beneficial for sustaining soil nutrient availability and biochemical activity under water-limited conditions.

### 4.2 Responses of soil organic carbon fractions to deficit irrigation and straw mulching

Soil organic carbon consists of multiple fractions with distinct physicochemical characteristics, and these fractions respond differently to deficit irrigation and straw mulching (Hu et al., 2023). In the present study, increasing irrigation deficit did not produce uniform changes across soil organic carbon fractions. LFOC, CPOC, and EOC exhibited relatively large variations, whereas FPOC remained comparatively stable (**Figure 6A**). This pattern suggests that water stress preferentially affects labile carbon fractions, which is consistent with findings in karst soils showing that active organic carbon pools are more sensitive to environmental changes (Zheng et al., 2014). Under the same deficit irrigation level, straw mulching did not simply increase SOC but changed the relative proportions of different carbon fractions within SOC, thereby regulating soil organic carbon structure (Sun et al., 2021; Ding et al., 2025). Gu et al. (2016) similarly found that long-term straw mulching exerted distinct effects on different soil organic carbon fractions. Further analysis indicated that under W1, CPOC and EOC and their proportions within SOC were relatively higher under F1, whereas both their contents and proportions declined with increasing irrigation deficit (**Figure 7**). Dong et al. (2018) also reported that straw mulching promoted the accumulation of POC and EOC under relatively adequate water supply, whereas this promoting effect weakened under limited water availability.

In contrast, LFOC accounted for a relatively higher proportion under F2 (**Figure 6B**). Although this result appears inconsistent with the commonly expected positive effect of straw input on labile organic carbon, labile organic carbon fractions are highly sensitive indicators and may respond differently to management practices. Zhang et al. (2020) reported that labile organic carbon fractions are early and sensitive indicators of soil quality changes. Li et al. (2016) showed that straw mulching had positive or no obvious effects on different labile organic carbon fractions during crop growth, indicating that straw mulching does not necessarily increase all active carbon fractions simultaneously. In addition, Liu et al. (2013) demonstrated that straw mulching favors the accumulation of heavy organic carbon fractions (HFOC), which may reduce the relative proportion of LFOC within SOC. Consequently, the higher proportional contribution of LFOC under F2 may reflect differences in soil organic carbon fraction redistribution rather than absolute carbon accumulation. All in all, the regulatory effect of straw mulching on soil organic carbon fractions varied with irrigation deficit intensity, with particularly pronounced responses observed in active carbon pools such as CPOC and EOC.

### 4.3 Pathways linking soil quality changes to maize yield

SQI provided a quantitative basis for explaining changes in integrated soil quality under deficit irrigation and straw mulching. Compared with individual soil indicators, SQI integrates information on soil nutrients, enzyme activities, and organic carbon fractions, thereby providing a more comprehensive evaluation of soil quality. Lenka et al. (2022) indicated that SQI constructed based on multiple indicators can be used to comprehensively evaluate agricultural soil quality and compare soil quality responses under different management practices. In the present study, the higher SQI under straw mulching treatments were generally consistent with higher standardized scores of soil nutrients, enzyme activities, and some labile organic carbon fractions (**Figure 7**). This suggests that the positive effect of straw mulching on integrated soil quality was not determined by changes in a single indicator, but was reflected in the integrated response of multiple soil indicators. Adak et al. (2023) also reported that residue retention under conservation agriculture increased SQI and that SQI was closely associated with system productivity.

On the basis of the changes in integrated soil quality, the WUE and IWUE results showed the performance of different treatments in terms of water saving and yield maintenance. In the present study, when the seasonal deficit irrigation amount was reduced from 2625 m³ ha^-^¹ to 2400 m³ ha^-^¹, W2F1 still maintained a grain yield similar to that of W1F1 and showed the highest WUE and IWUE (**Table 1**). This indicates that the advantage of W2F1 was not only reflected in maintaining a relatively high yield, but also in achieving higher water productivity under lower irrigation input. Therefore, under the semi-arid field conditions of this study, moderate deficit irrigation combined with straw mulching could improve water productivity while maintaining yield. However, when the irrigation deficit intensified to W3, maize yield decreased, and the advantages in WUE and IWUE were also weakened, indicating that excessive deficit irrigation may exceed the compensatory capacity of straw mulching. Zou et al. (2021) also reported that appropriately controlled deficit irrigation can maintain relatively high maize yield while reducing irrigation water use in semi-arid environments. However, when the irrigation deficit is excessive, insufficient water supply can restrict maize growth and yield formation, resulting in yield reduction (Bayisa et al., 2021).

Correlation analysis, PCA, and SEM results showed that maize yield response could not be explained only by a single nutrient indicator, but was closely related to enzyme activity, labile organic carbon fractions, and integrated soil quality. Among these variables, CPOC and enzyme activity were closely associated with yield performance (**Figures 8**, **9**). This suggests that, under the short-term field management conditions of this study, changes in labile carbon pools and enzyme-mediated nutrient transformation processes contributed to yield response, in addition to soil nutrient contents. Compared with SOC, CPOC is a relatively active organic carbon fraction and is usually more sensitive to straw input, mulching management, and soil moisture changes in many field management studies. Sainju et al. (2022) found that particulate organic carbon was more strongly associated with crop yield than total nitrogen or available nutrient indicators in long-term dryland systems. This finding is consistent with the relatively close relationship between CPOC and maize yield observed in the present study and also supports the use of CPOC as a sensitive indicator reflecting management-induced changes in soil carbon dynamics and their relationship with crop productivity.

**Figure 10:**
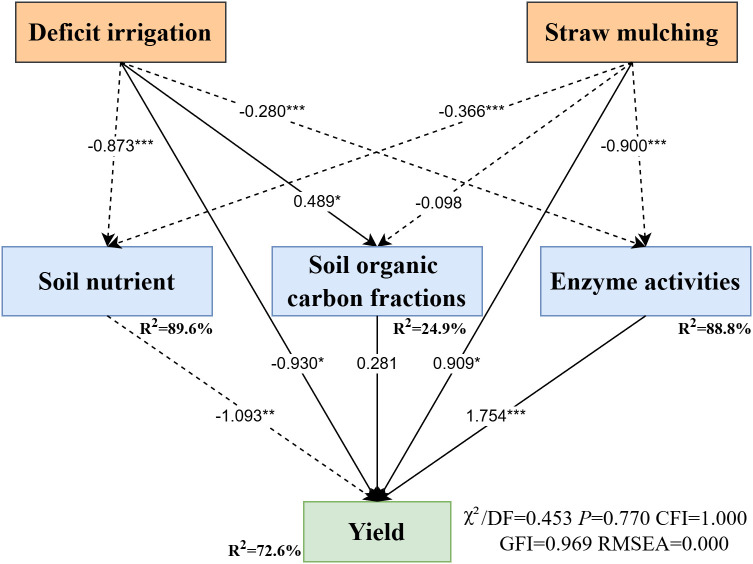
Structural equation model (SEM) illustrating the effects of deficit irrigation and straw mulching on maize yield. The solid and dashed arrows indicate positive and negative relationships, respectively. The number on each arrow indicates the normalized path factor and R² represents the proportion of variance explained by the model. *, **, and *** indicate significance at *P* < 0.05, *P* < 0.01, and *P* < 0.001, respectively. χ²/DF indicates the chi-square to degrees of freedom ratio, *P* indicates the significance level, RMSEA represents the root mean square error of approximation, CFI represents the comparative fit index, GFI represents the goodness of fit.

SEM results showed that deficit irrigation had a negative direct effect on yield, whereas straw mulching had a positive direct effect on yield. Meanwhile, deficit irrigation significantly affected soil nutrients, enzyme activity, and soil organic carbon fractions, whereas straw mulching significantly affected soil nutrients and enzyme activity (**Figure 10**). Among the soil process variables, enzyme activity had a significant positive direct effect on maize yield, and soil organic carbon fractions also showed a positive effect trend. This suggests that yield maintenance under straw mulching was not only related to improved soil moisture conditions, but also to enzyme-mediated nutrient transformation and changes in organic carbon fractions. Guan et al. (2020) found that straw management promoted straw decomposition and nutrient release, indicating that straw input can affect soil nutrient supply through organic matter decomposition.

Deficit irrigation and straw mulching altered soil nutrients, enzyme activities, and organic carbon fractions, and these changes were jointly reflected in the integrated response of SQI and were associated with maize yield and water productivity. Compared with individual nutrient indicators, SQI, enzyme activity, and labile organic carbon fractions provided a more integrated basis for explaining the relationships among soil quality changes, water-saving effects, and yield maintenance in semi-arid maize production.

However, this study was conducted during one growing season, did not include a full irrigation control, and focused on the 0–20 cm soil layer. Therefore, the results mainly reflect relative differences among deficit irrigation levels and surface soil responses, rather than the absolute water-saving effect or yield penalty compared with full irrigation. Because soil quality responses and SEM pathways may be affected by experimental duration, interannual climatic variation, and soil sampling depth, future long-term field experiments including a full irrigation control and deeper soil profile observations are needed to verify the stability of these effects under different climatic conditions.

## 5 Conclusion

This study evaluated the effects of deficit irrigation and straw mulching on soil nutrients, enzyme activities, soil organic carbon fractions, SQI, maize yield, WUE, and IWUE in semi-arid maize field. The results showed that increasing irrigation deficit generally reduced soil nutrient contents, several enzyme activities, labile organic carbon fractions, and maize yield, whereas straw mulching partly alleviated these negative effects and improved integrated soil quality. W1F1 showed the highest SQI and had relatively high scores for several nutrient, enzyme activity, and organic carbon indicators, indicating its advantage in maintaining integrated soil quality. However, W2F1 maintained a grain yield comparable to W1F1 under a lower irrigation amount and achieved the highest WUE and IWUE, showing its advantage in water saving and yield maintenance.

Correlation analysis, PCA, and SEM further indicated that maize yield was closely associated with enzyme activity, labile organic carbon fractions, and integrated soil quality. The SEM results suggested that yield responses were related to both the direct effects of deficit irrigation and straw mulching and changes in soil quality properties. Overall, W1F1 was more favorable for maximizing integrated soil quality, whereas W2F1 provided the best balance between yield maintenance, water saving, and soil quality preservation. Therefore, moderate deficit irrigation combined with straw mulching can be considered a feasible water-saving strategy for sustainable maize production in semi-arid environments.

## Data Availability

The original contributions presented in the study are included in the article/**Supplementary Material**. Further inquiries can be directed to the corresponding authors.
