# Supplementary material for: Deficit irrigation combined with straw mulching maintains maize yield and improves soil quality in semi-arid environments
Source: Front Plant Sci. 2026 Jun 15;17:1823857. doi: 10.3389/fpls.2026.1823857 (PMC13310724; doi:10.3389/fpls.2026.1823857)
Supplement: Supplementary file 1 [file DataSheet1.docx]

Supplementary Material

# Supplementary Determination of Soil Enzyme Activity

## Urease

Sodium Phenolate-Sodium Hypochlorite Colorimetric Method

### Preparation of Reagents

(1) pH 6.7 citrate solution:

Dissolve 184 g of citric acid in 300 mL of distilled water. Separately, dissolve 147.5 g of potassium hydroxide in water. Combine the two solutions, adjust the pH to 6.7 using a 10 N sodium hydroxide/1 N sodium hydroxide solution, and dilute with water to 1 L.

(2) Sodium Phenolate Solution:

Weigh 62.5 g of phenol and dissolve it in a small amount of ethanol. Add 2 mL of methanol and 18.5 mL of acetone, then dilute with ethanol to 100 mL (Solution A). Store in the refrigerator. Weigh 27 g of sodium hydroxide and dissolve it in 100 mL of water (Solution B). Store in the refrigerator. Before use, combine solutions A and B, and dilute with distilled water to 500 mL for later use.

(3) Sodium hypochlorite solution:

Dilute the preparation with water to a concentration of 0.9% active chlorine; the solution is stable. Transfer 12.5 mL of the 8% sodium hypochlorite solution and dilute to 1 L.

(4) 10% urea solution:

Dissolve 10 g of urea in 100 mL of water.

(5) Standard nitrogen solution:

Accurately weigh 0.4717 g of ammonium sulfate, dissolve it in water, and dilute to 1 L. This yields a standard solution containing 0.1 mg of nitrogen per mL (100 ppm nitrogen standard).

**Plotting the Standard Curve:** Take 0, 0.2, 0.4, 0.6, 0.8, 1, 1.2, 1.6, and 2 mL of nitrogen standard solution into 50 mL graduated test tubes, add distilled water to a total volume of 10 mL, then add 4 mL of sodium phenolate solution and 3 mL of sodium hypochlorite solution, shaking well immediately after each addition to obtain a series of standard curves with concentrations of 0, 0.4, 0.8, 1.2, 1.6, 2, 2.4, 3.2, and 4 mg/L. After 20 minutes, make up to 50 mL and allow the color to develop. Measure the absorbance at 578 nm using a spectrophotometer within 1 hour.

### Procedure

(1) Weigh 1 g of soil and weigh into a 30 mL plastic bottle. Add 1 drop of toluene, seal the bottle tightly, and shake gently for 15 minutes. Add 4 mL of citrate buffer (pH 6.7), followed by 2 mL of 10% urea solution, and mix thoroughly. Incubate in a 37°C incubator for 24 hours.

(2) After incubation, filter the suspension using qualitative filter paper.

(3) Transfer 0.2 mL of the filtrate to an 80-mL plastic bottle, dilute to 10 mL with distilled water, then add 4 mL of sodium phenolate solution, followed immediately by 3 mL of sodium hypochlorite solution. Shake the mixture well immediately after adding each reagent. After 20 minutes, dilute the mixture to 50 mL and measure within 1 h.

(4) Measure the absorbance at a wavelength of 578 nm. Urease activity is calculated as the difference between the absorbance of the sample and that of the control sample. The amount of ammonia nitrogen is determined based on the standard curve.

### Calculation of Results

Urease activity is expressed as the mass (mg) of NH₃-N in 1 g of soil after 24 hours.

Urease = a · V · n / m

a is the NH₃-N concentration (mg/mL) determined from the standard curve;

V is the volume of the color-developing solution (50 mL); V is the final volume after dilution for colorimetric measurement;

n is the dilution factor (50); k is the ratio of the total extract to the volume of the color-developing solution drawn;

m is the weight of the dried soil (g). Fresh soil mass multiplied by 1 minus the moisture content.

## Catalase

Potassium Permanganate Titration Method

### Preparation of Reagents

1. 1.5 mol/L H₂SO₄ solution:

Measure 83.3 mL of concentrated sulfuric acid and dilute to 1000 mL; store in the refrigerator.

(2) 0.02 mol/L potassium permanganate solution:

Weigh 3.26 g of potassium permanganate, add to 400 mL of water, boil gently for 15 minutes, cool, and make up to 500 mL. Store away from light; standardize with 0.1 mol/L oxalic acid solution when needed.

(3) 0.3% hydrogen peroxide solution:

0.1 mol/L oxalic acid solution: Weigh 3.334 g of analytical-grade H₂C₂O₄·2H₂O, dissolve in distilled water, and make up to 250 mL;

3% H₂O₂ aqueous solution: Take 25 mL of 30% H₂O₂ solution, dilute to 250 mL, store in the refrigerator, and titrate with 0.1 mol/L KMnO₄ solution when needed.

### Procedure

Weigh 2 g of air-dried soil, place it in a 100-mL conical flask, and add 40 mL of distilled water and 5 mL of 0.3% hydrogen peroxide solution. Shake the conical flask on a reciprocating shaker for 20 minutes. Then add 5 mL of 3 N sulfuric acid to stabilize the decomposing hydrogen peroxide. Filter the suspension in the flask using slow-flow filter paper. Pipette 25 mL of the filtrate and titrate with potassium permanganate until a pale pink endpoint is reached.

### Calculation of Results

Catalase = (A – B) × T / soil sample mass

A is the volume (ml) of potassium permanganate consumed in titrating 25 mL of the hydrogen peroxide mixture;

B is the volume (ml) of potassium permanganate consumed in titrating the soil filtrate;

T is the correction factor for the titration value of potassium permanganate.

## Phosphatase

Disodium Phenyl Phosphate Colorimetric Method

### Preparation of Reagents

(1) Buffer solutions:

(a) Acetate buffer (pH 5.0): A 0.2 mol L⁻¹ acetic acid solution was prepared by diluting 11.55 mL of glacial acetic acid (95%) to 1 L with distilled water. A 0.2 mol L⁻¹ sodium acetate solution was prepared by dissolving 16.4 g of C₂H₃O₂Na (or 27 g of C₂H₃O₂Na·3H₂O) in water and diluting to 1 L. Then, 14.8 mL of the acetic acid solution and 35.2 mL of the sodium acetate solution were mixed and diluted to 1 L.

(b) Citrate–phosphate buffer (pH 7.0): A 0.1 mol L⁻¹ citric acid solution was prepared by dissolving 19.2 g of C₆H₈O₇ in water and diluting to 1 L. A 0.2 mol L⁻¹ Na₂HPO₄ solution was prepared by dissolving 53.63 g of Na₂HPO₄·7H₂O (or 71.7 g of Na₂HPO₄·12H₂O) in water and diluting to 1 L. Then, 6.4 mL of the citric acid solution and 43.6 mL of the Na₂HPO₄ solution were mixed and diluted to 100 mL.

(c) Borate buffer (pH 9.6): A 0.05 mol L⁻¹ borax solution was prepared by dissolving 19.05 g of borax in water and diluting to 1 L. A 0.2 mol L⁻¹ NaOH solution was prepared by dissolving 8 g of NaOH in water and diluting to 1 L. Then, 50 mL of the borax solution and 23 mL of the NaOH solution were mixed and diluted to 200 mL.

(2) Substrate solution (0.5% disodium phenyl phosphate):

Disodium phenyl phosphate (2.5 g) was dissolved and diluted to 500 mL using the corresponding buffer solution.

(3) 2,6-dichloro-4-dibromoquinone chlorimide reagent:

0.125 g of reagent was dissolved in 10 mL of 96% ethanol and stored in a brown bottle at 4°C.

(4) Toluene

(5) Aluminum sulfate solution (0.3%):

Prepared by dissolving aluminum sulfate in water to obtain a 0.3% solution.

(6) Phenol standard solution:

A stock solution was prepared by dissolving 1 g phenol in distilled water and diluting to 1 L. A working solution (0.01 mg mL⁻¹) was prepared by diluting 10 mL of the stock solution to 1 L.

**Standard curve preparation:** Aliquots of 0, 1, 3, 5, 7, 9, 11, and 13 mL of phenol working solution were transferred into 50 mL volumetric flasks. Each flask received 5 mL borate buffer and 4 drops of 2,6-dichloro-4-dibromoquinone chlorimide reagent. After color development, the solutions were diluted to volume. After 30 min, absorbance was measured at 660 nm. A standard curve was constructed using phenol concentration as the x-axis and absorbance as the y-axis.

### Procedure

Weigh 5g of the soil sample in a 200 mL Erlenmeyer flask. Then, 2.5 mL of toluene was added and the mixture was shaken gently for 15 min.

After that, 20 mL of 0.5% disodium phenyl phosphate solution was added. The substrate solution was prepared with different buffers depending on enzyme type: acetate buffer for acid phosphatase, citrate–phosphate buffer for neutral phosphatase, and borate buffer for alkaline phosphatase.

The mixture was incubated at 37°C for 24 h. After incubation, 100 mL of 0.3% aluminum sulfate solution was added, and the suspension was filtered.

An aliquot of 3 mL of filtrate was transferred into a 50 mL volumetric flask. Color development was carried out following the same procedure as for the standard curve. When borate buffer was used, a blue color developed, and absorbance was measured at 660 nm.

### Calculation of Results

Phosphatase activity was expressed as mg phenol released per g soil over 24 h.

$$\text{Phosphatase}=\left( a_{\text{sample}}-a_{\text{soil blank}}-a_{\text{substrate blank}} \right)\times V\times\frac{n}{m}$$

$a_{\text{sample}}$is the phenol content derived from the sample absorbance;

$a_{\text{soil blank}}$ is the phenol content of the soil-free control;

$a_{\text{substrate blank}}$ is the phenol content of the substrate-free control;

V is the volume of the color-developed solution;

n is the dilution factor;

m is the oven-dry weight of soil.

## Protease

Ninhydrin Colorimetric Method

### Preparation of Reagents

1% casein solution, prepared with 0.2 M phosphate buffer at pH 7.4; 0.05 mol L⁻¹ sulfuric acid solution; 20% sodium sulfate solution; 2% ninhydrin solution, prepared with acetone;

Toluene; Glycine; Anhydrous ethanol; Glycine standard solution: Accurately weigh 0.1000 g of glycine, dissolve it in water, and dilute to 1000 mL. This gives a 0.02 mg mL⁻¹ amino nitrogen standard solution.

**Plotting the Standard Curve:** Accurately transfer 0, 0.5, 1.0, 2.0, 3.0, 4.0, and 5.0 mL of the 0.02 mg mL⁻¹ glycine standard solution into seven 50 mL volumetric flasks. Add 1 mL of ninhydrin solution to each flask. Rinse the neck of the flask and heat in a boiling water bath for 10 min. Remove the flasks, cool them, and dilute to volume with water. Mix well and measure the absorbance at 500 nm using a UV–visible spectrophotometer. Plot the calibration curve using amino nitrogen concentration as the x-axis and absorbance as the y-axis.

### Procedure

Weigh 4 g of air-dried soil into a 50 mL plastic centrifuge tube. Add 20 mL of 1% casein solution and 1 mL of toluene. Tighten the cap, mix well, and incubate at 30°C for 24 h.

After incubation, precipitate the protein. Transfer 2 mL of the supernatant into a 50 mL volumetric flask. Add 1 mL of ninhydrin solution, rinse the neck of the flask, and mix well. Heat in a boiling water bath for 10 min. Remove the flask, dilute to volume with water, and mix well.

Measure the absorbance at 500 nm using a UV–visible spectrophotometer. A substrate-free control and a soil-free control should be prepared for each soil sample.

### Calculation of Results

Protease activity is expressed as the amount of amino nitrogen released per gram of soil.

$$\text{Protease activity}=\frac{C\times V\times D}{m}$$

C is the amino nitrogen concentration obtained from the standard curve;

V is the final volume of the color-developed solution;

D is the dilution factor;

m is the soil mass.

## Cellulase and Sucrase

3,5-dinitrosalicylic Acid Colorimetric Method

### Preparation of Reagents

(1) 3,5-Dinitrosalicylic Acid Solution:

Weigh 0.5 g of dinitrosalicylic acid, dissolve it in 20 mL of 2N sodium hydroxide and 50 mL of water, then add 30 g of potassium sodium tartrate, and dilute with water to 100 mL.

(2) pH 5.5 phosphate buffer:

1/15 M disodium hydrogen phosphate (dissolve 23.876 g of disodium hydrogen phosphate·12H₂O in 1 L of distilled water; or dissolve 11.867 g of disodium hydrogen phosphate dihydrate in 1 L of distilled water)

Add 0.5 mL of 1/15 M potassium dihydrogen phosphate solution (9.078 g of potassium dihydrogen phosphate dissolved in 1 L of distilled water) to 9.5 mL of the solution to prepare the final solution.

(3) 8% sucrase solution:

Dissolve 80 g of sucrase in 1000 mL of water

(4) Toluene

(5) Standard glucose solution:

Pre-dry the glucose at 80°C until constant weight. Then dissolve 50 mg in 50 mL of distilled water to prepare the standard glucose solution.

**Plotting the Standard Curve:** Take 0, 0.5, 1, 1.5, 2, 3, and 4 mL of the glucose working solution and inject them into 50 mL graduated test tubes, respectively. Perform the color development using the same method as for determining sucrase activity. After colorimetry, plot the standard curve with absorbance as the vertical axis and glucose concentration as the horizontal axis.

### Procedure

Weigh 1 g of soil and place it in a 30-mL plastic bottle. Add 3 mL of 8% sucrase solution, 1 mL of pH 5.5 phosphate buffer, and 1 drop of toluene. Shake the mixture well, then place it in an incubator and incubate at 37°C for 72 hours.

After the incubation period, remove the bottle and filter the contents quickly using qualitative filter paper. Pipette 50–100 μL of the filtrate into a 50 mL volumetric flask, add 3 mL of 3,5-dinitrosalicylic acid, and heat in a boiling water bath for 5 minutes. Immediately transfer the flask to a stream of tap water to cool for 3 minutes. The solution turns orange-yellow due to the formation of 3-amino-5-nitrosalicylic acid. Finally, dilute to 50 mL with distilled water and measure the absorbance at a wavelength of 508 nm using a spectrophotometer.

### Calculation of Results

Cellulase/Sucrase activity is expressed as the mass of glucose (mg) in 1 g of soil after 72 hours:

Cellulase/Sucrase = a · V · n / m

a is the glucose concentration (mg/mL) determined from the standard curve;

V is the volume of the color development solution (50 mL);

n is the dilution factor;

m is the weight of the dried soil (g).
